# Energetic and Economic Evaluation of Zero-Waste Fish Co-Stream Processing

**DOI:** 10.3390/ijerph18052358

**Published:** 2021-02-28

**Authors:** Kęstutis Venslauskas, Kęstutis Navickas, Marja Nappa, Petteri Kangas, Revilija Mozūraitytė, Rasa Šližytė, Vidmantas Župerka

**Affiliations:** 1 Institute of Energy and Biotechnology Engineering, Vytautas Magnus University, K. Donelaičio g. 58, LT-44248 Kaunas, Lithuania; kestutis.navickas@vdu.lt (K.N.); vidmantas.zuperka1@vdu.lt (V.Ž.); 2 VTT Technical Research Centre of Finland Ltd., 02150 Espoo, Finland; marja.nappa@vtt.fi (M.N.); petteri.kangas@vtt.fi (P.K.); 3 SINTEF Ocean, 7010 Trondheim, Norway; Revilija.Mozuraityte@sintef.no (R.M.); rasa.slizyte@sintef.no (R.Š.)

**Keywords:** salmon, co-stream, fish protein hydrolysate, biogas, modelling

## Abstract

This study evaluates the possibility of recovery of high-quality valuable fish oil and proteins from fish co-streams by traditional means or a combination of several technologies. A techno-economically feasible and sustainable zero-waste process is needed for full utilisation of this co-stream’s potential. This study aims to determine the energy efficiency and economic feasibility of four different zero-waste bio-refineries based on salmon filleting co-streams. The study covers four concepts: (I) biogas and fertiliser production from salmon co-streams, (II) fish silage production, (III) thermal processing of salmon co-streams for producing oil, protein concentrate, and meal, and (IV) novel two-stage thermal and enzymatic process for producing high-quality oil and protein hydrolysate, while the solid residue is converted to biogas and fertilisers. Monte Carlo simulation is used to evaluate uncertainties in economic evaluation. The results show that the two-stage processing of fish co-streams leads to recovery of both high-quality marine oil and proteins, showing the largest profitability and return on investment during the economic analysis. It is a more tempting option than the currently used thermal treatment or traditional silage processes. The possibility of producing food-grade fish protein hydrolysate is the biggest benefit here. Concepts studied are examples of zero-waste processing of bioproducts and illustrate the possibilities and benefits of fully utilising the different fractions of fish as fillets, oil, protein, fertilisers, and energy production.

## 1. Introduction

The world is concerned about the use of natural resources and the deployment of sustainable practices and goals. Greater attention should be given to the implementation of sustainable agriculture, fisheries, and aquaculture, solving several challenges including water scarcity and food waste problems [1]. Fish processing into different products on an industrial level generates a significant quantity of co-streams, which are often considered as low-value products or even can be wasted without any attempt to recover. Such a point of view is unattractive in terms of circular economy [2].

Aquaculture is the fastest growing producer of animal protein in the world. The UN Food and Agriculture Organization (FAO) estimates that by 2030 aquaculture will account for 2/3 of the seafood produced [3]. Salmon is one of the most popular aquaculture fish in the world, and Norway is currently the world’s largest producer of farmed salmon. It is estimated that salmon aquaculture will grow and reach up to 5·10^6^ tonnes in 2050, which means a 4–5 times increase from the current level [4]. Due to this, it is important to estimate and find the most optimal and sustainable way of utilising salmon processing co-streams, taking into account that this fish as well as co-streams are not seasonal goods and can be generated and supplied to the market year-round.

It is estimated that up to 50–75% of fish end up as co-streams in the seafood industry [5]. In Norway approximately 77% of fish processing co-streams are utilised, where 72% of the utilised amount is used for fish meal and silage production and ~13% for production of human consumption products [6,7]. In many countries, due to the lack of specialised infrastructure, co-streams are wasted or sent directly for animal feed without any attempt to recover the valuable components. However, these co-streams can be upgraded into more valuable products like w:3 rich oils, gelatine, and marine proteins [8,9]. Enzymatic and thermal treatment of animal-based co-streams lead to oil, protein, and sediment fractions, where the latter contain lipids, insoluble proteins, and the rest of the bones and scales [10,11,12,13]. The remaining sediments after the recovery of valuable products from fish co-streams contain a high concentration of nitrogen (N), phosphorus (P), and calcium (Ca) [14,15] and could be used for biogas production and bio-fertiliser generation [16,17]. Generated biogas has various applications, such as purification to biomethane [18,19], generation of biohydrogen [20], biomethanol [21,22,23,24,25], or used in fuel cells for power generation [26] and chemicals production [27]. The obtained digestate after anaerobic digestion could be used as an organic fertiliser for the fields of the farm [28,29,30,31]. The composition of the mixture of fish co-streams with cattle manure should be formed as nutrients for plant fertilisation [32,33]. Co-digestion of fish co-streams with cattle manure could improve the energy efficiency of biogas plant and fertilisation quality of the final digestate [34,35]. Energy balance of the system includes the total energy input, which comprises direct and indirect energy inputs [36] of the full cycle covering soil cultivation, biomass production, anaerobic digestion, and fertilisation of soil by the digestate [37].

Biogas generation from animal by-products supports the 34% target of the European Union (EU 27) for the contribution of renewable sources from final energy consumption by 2030 [38]. The use of biomass for energy generation reduces the greenhouse gas emissions compared to the use of fossil fuels [39,40] with a special focus on green biomass [41] such as forest residues [42], maize [43], or other biomass [44].

There is also a lack of optimal technological solutions for the recovery of high-quality valuable fish oil and proteins from fish co-streams for human consumption. Traditionally enzymatic hydrolysis and/or thermal treatments are used for processing of fish co-streams [5,12]. The new technological concept presented by Slizyte et al. [45] produces high-quality fish oil and protein-rich fractions more sustainably and profitably. A techno-economically feasible and sustainable zero-waste process is needed to reach full utilisation of this co-stream’s potential [46]. It is expected that new innovative food processing technologies and products will help to contribute to climate change mitigation [2,47].

The aim of the zero-waste biorefinery is directed to 100% utilisation of valuable fish filleting co-streams by producing high-value products (biomaterials, nutraceutical, cosmetics, and ingredients for food and feed) [48], followed by utilisation of the remaining residue for co-generation of energy and soil fertilisers. This study aims to determine the energy efficiency and economic feasibility of four different zero-waste bio-refineries based on salmon fileting co-streams.

## 2. Materials and Methods

An energetic and economic evaluation of four technological concepts for the processing of salmon processing co-streams is presented in this study: (I) biogas and fertiliser production from salmon co-streams, (II) fish silage production, (III) thermal processing of salmon co-stream for producing oil, protein concentrate, and meal, and (IV) novel two-stage thermal and enzymatic process for producing high-quality oil and protein hydrolysate, while the solid residue is converted to biogas and fertilisers.

**Figure 1:**
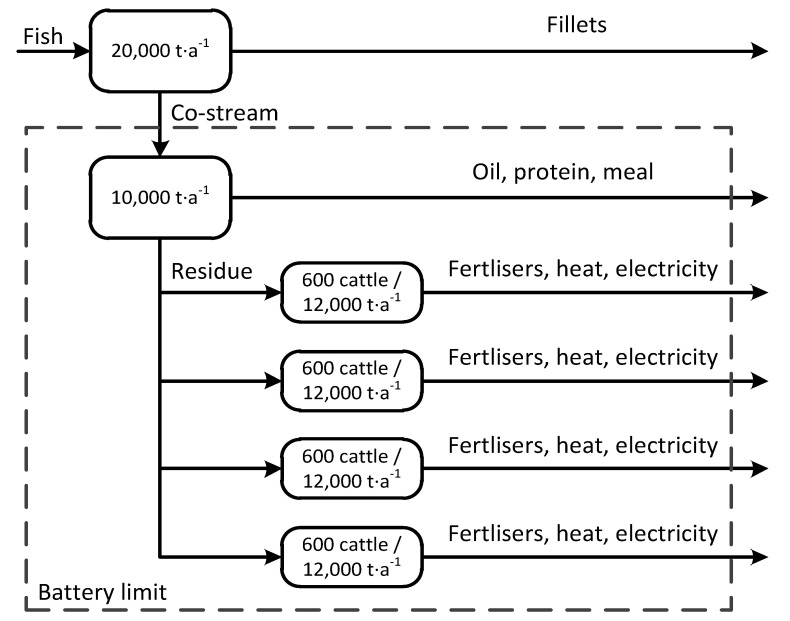
Battery limits of the modelled system in this study.

Fish aquaculture and slaughterhouses are situated near the shore in Northern Europe. More than 1,300,000 t of salmon is annually processed in slaughterhouse resulting in more than 400,000 t co-stream flows (heads, backbones, and viscera) [6]. The chosen battery limits for the modelled system are given in Figure 1.

The fish co-stream processing plant is situated next to the slaughterhouse. Annual operation time of this plant is ~3000 h (in concepts II, III, and IV) indicating operation in one shift [49]. Heads, backbones, and viscera from the processes (of concepts I, III, and IV) are transported to four farms. The approximate road distance between the fish processing plant and farms (the digestion facilities) is assumed to be 100 km (by 24 t lorry). In each farm, the additional fertilisation of arable land (600 ha) intended for summer barley growing is needed. Barley nutrient requirements are N110, P40, and K80 kg·ha^−1^. In addition, cattle of 600 milking cows produce 12,000 t of manure annually in each of the farms. When biogas is produced (in concepts I and IV), the micro-turbine is utilised for combined heat and power production.

Economic evaluation was conducted with stochastic simulation, here Microsoft Excel (Microsoft Corporation, Redmond, WA, USA) and @Risk (Palisade Corporation, Ithaca, NY, USA) [50] were used as tools. @Risk is an add-in tool to Ms Excel that lets you analyse uncertainties using Monte Carlo simulation. Energetic evaluation was based on the process simulation by SimaPro (©PRé Sustainability B.V., Amersfoort, The Netherlands) [51]. Key input parameters were obtained from the literature. Next, evaluated concepts are presented in more detail.

### 2.1. Processing Fish Co-Stream to Biogas and Fertilisers

**Figure 2:**
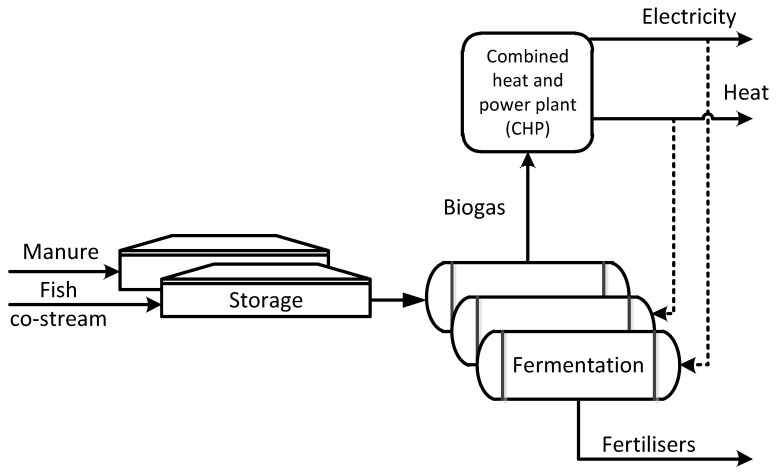
Processing the fish co-stream to biogas and fertilisers (I).

The fish co-stream was considered here as residue and is used for biogas and fertiliser production, see Figure 2. The residue is transported from the slaughterhouse to the farm by lorry. The system assessed was the anaerobic digestion of fish co-stream and cattle manure on a regional scale, while co-digestion of other feedstock was not considered. Liquid manure and fish co-stream were mixed at a ratio 5:1 by raw material mass together before feeding to the digester. Anaerobic digestion was assumed to be a wet, single-stage, continuously fed process operating at mesophilic temperature (+38 °C) and an organic loading rate of 2 kg volatile solids (VS) m^3^·d^−1^ with total solids (TS) content at the input of 12.1 wt.%. The volume of the anaerobic digester was 2300 m^3^. Parameters associated with the fish backbones and animal manure treated within the anaerobic digestion plants were a biogas yield of 88 m^3^·t^−1^ of mixture and methane content of 65 v.%. The same parameters were used for biogas plant for treating the residues in concept IV.

### 2.2. Production of Fish Silage and Fertilisers 

**Figure 3:**
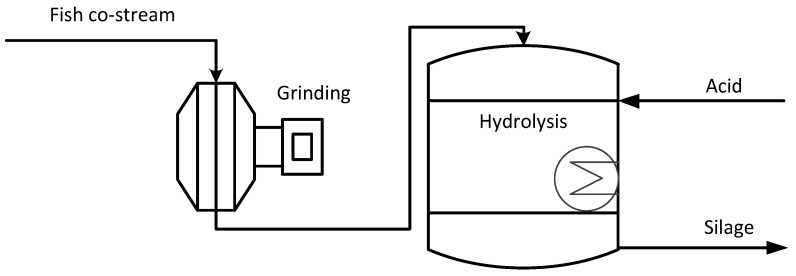
Fish silage process (II).

Fish silage to be used as an animal meal can be considered as the simplest processing method for fish co-streams, see Figure 3. The co-stream is processed by acidification and preserved in silage form [5,6,13,52]. First, the co-stream is grinded to decrease the particle size, and acid (formic acid) is added to reduce the pH to 3.5–4.0. The silage is stored in containers for several days or weeks. Low pH inhibits microbial activity but enables the activity of endogenous proteolytic enzymes from fish to hydrolyse, which liquefies material. Elevated temperature (to 50 °C) speeds up the reaction. Silage can be used as liquefied feed, typically for pigs.

As the entire fish co-stream is used as a meal, no residue is left for biogas production. In this concept the farm land (totally 600 ha) was divided into two areas where 400 ha fertilised with cattle manure with additional P-fertilisers, and another area of 200 ha fertilised with mineral fertilisers only. The application rate of cattle manure was 30 t·ha^−1^ and additionally 484 kg·ha^−1^ of mixed fertilisers (ammonium nitrate; ammonium di-hydrophosphate; potassium chloride). The area of 200 ha was fertilised by application of 534 kg·ha^−1^ of mixed fertilisers (ammonium nitrate; ammonium di-hydrophosphate; potassium chloride). Mineral fertiliser was transported 100 km from the supplier to the farm by a lorry with the transportation capacity of 24 t. The storage of 500 m^2^ was designed for fertilisers at the farm. From storage place, fertilisers were transported to the fields which were 10 km away by a tractor (155 HP) with 13 t trailer. Liquid manure was stored in an 8000 m^3^ tank, which is littered by injecting spreader with a capacity of 7 m^3^ and 12 m width. The same specification was also applied when concept III is analysed.

### 2.3. Production of Fish Oil, Protein Concentrate and Meal

**Figure 4:**
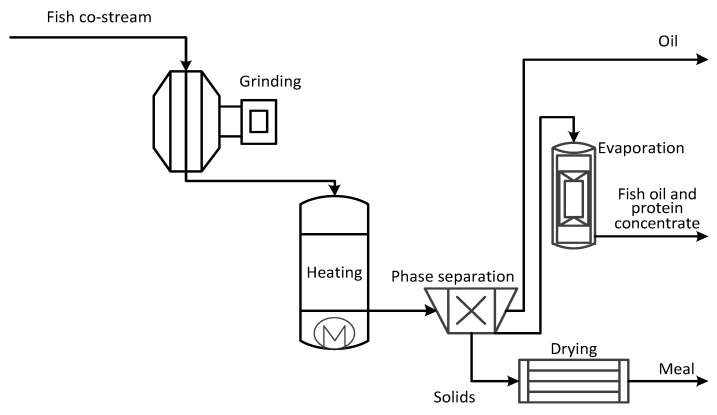
Production of oil and fish protein concentrate (III).

This concept covers the production of fish oil and protein concentrate (FPC), see Figure 4. The fish co-stream was minced to produce homogenous feed for the process. Thermal treatment (>90 °C) was used to fractionate the oil from the raw material, after which the individual phases were separated. For example, a tri-canter can be applied here. The separated phases were oil, protein-rich stick water, and meal. The aqueous phase (e.g., stick water) was evaporated to 50 wt.% concentration, and the meal was dried. The fish protein concentrate process is a common procedure in the industry [53]. Obtained products are typically used as animal feed. 

As fish co-stream is fully utilised in this concept, and no residue is considered here. The fertilisation of arable land is conducted similarly as for concept II: manure and additional mineral fertilisers are applied.

### 2.4. Production of Premium Oil and Fish Protein Hydrolysate and Utilising Residues for Biogas Production

**Figure 5:**
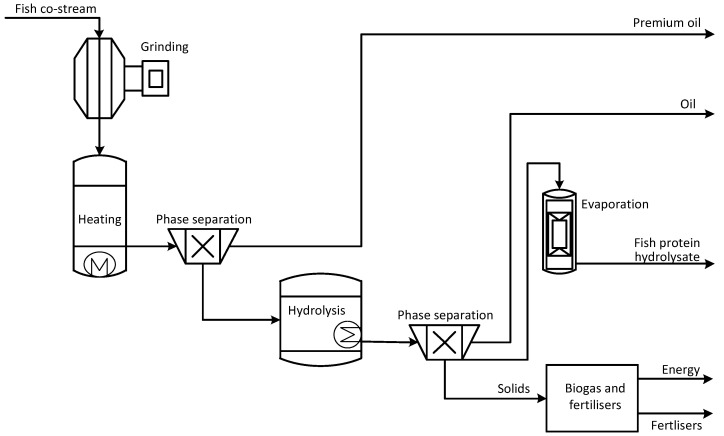
Production of premium oil and fish protein hydrolysate: two-stage processing (IV).

Production of premium oil and protein-rich hydrolysate was conducted in a novel two-stage process, see Figure 5. The principles of this process are given in the work of Slizyte et al. [45]. The process differs from the thermal process (concept III) as it uses a gentler, mild thermal extraction during the first stage to obtain premium quality oil. Thermal extraction also minimizes the amount of oil in the following enzymatic process. From mild thermal extraction, the defatted fraction continued to enzymatic treatment. Enzymes were inactivated by heating after the enzymatic hydrolysis. The phase separator was applied to separate the oil, aqueous fish protein hydrolysate, and solids. The aqueous phase was evaporated to 50 wt.% concentration or dried. The aim of the gentle processing of the fish co-stream is to increase the quality and amount of value-added products and even utilise them as food instead of feed [45,54].

The solid residue was transported to the farm. When concept IV is considered the arable land was fertilised with a digestate obtained after anaerobic co-digestion of mixtures from fish backbones and cattle manure. However, the volume of residue was much smaller than in concept I, and so additional mineral fertilisers are needed.

## 3. Results

### 3.1. Material and Energy Balance

Material and energy balances of fish co-stream processing are adapted from the literature [49] (Table 1) and are presented per tonne of fish co-stream raw material. The typical values of the biogas plant are shown in Table 2, where the figure is collected from [55]. This balance date is applied for the evaluation of energetic and economic performances of four different fish co-stream processing technologies.

### 3.2. Energetic Analysis

When fish co-streams were analysed, high concentrations of nitrogen (62 g·kg^−1^) and phosphorus (13 g·kg^−1^) were found in backbones after salmon filleting (Table 3). Due to this, the co-digestion of salmon backbones and cattle manure was considered optimal for improving the efficiency of biogas plant and fertilisation quality of digestate. Mixing liquid (6 wt.% TS) cattle manure with dry (42 wt.% TS) fish backbones in 1:5 ratio was desirable for anaerobic digestion TS concentration (5–20 wt.% TS) [56].

It was assumed that the digestate generated from the biogas plants was compliant with the Publicly Availably Specification (PAS) 110 [57] and the Anaerobic Digestate Quality Protocol [58], and can therefore be considered as a product and utilised as a fertiliser within agriculture, forestry, or soil/field grown horticulture. The whole digestate was transported to the fields for fertilisation of barley. Transportation of digestate was undertaken using a tractor with a capacity of 7 m^3^. Tractor velocity when loaded was 15 km·h^−1^ and was 25 km·h^−1^ when empty. Distances for transporting digestate to the nearest arable land were assumed to be 10 km (arable land belonging to the farm).

At the biogas plant, a storage place of 500 m^2^ was modelled to store delivered fish backbones. Storage conditions were at ambient temperatures (+10 °C). The energy balance of the analysed system can be expressed as the difference between input and output and can be defined by the equation:

E_final_ = E_BM_ − E_input_ (1)

where E_final_ is final useful energy (MJ·ha^−1^); E_BM_ is the energy potential of biomass (MJ·ha^−1^), and E_input_ is the energy input of the system (MJ·ha^−1^). The energy potential of biomass was defined as potential biogas production from cattle manure and fish backbones and was determined according to the methodology explained by Navickas et al. (2013) [59] based on farm land area. According to the (unpublished) laboratory experiments, the biogas yield from the mixture of cattle manure and salmon backbones was considered 88 m^3^·t^−1^ from feedstock with methane concentration of 64.9 v.% in the biogas.

Various equipment and machinery are used for technological operations and processes; therefore, the total energy input can be expressed by the equation:

(2) $$E_{\mathrm{input}}=\overset{i}{\underset{1}{\sum }}E_{\mathrm{TEi}}$$

where E_TEi_ is total energy inputs (MJ·ha^−1^), and i is the number of technological operations/processes. The methodology of energy input for technological operations was presented and described previously in [37,60].

Total energy input is defined as direct and indirect energy input of the technological operation:

E_TEi_ = E_di_ + E_indi_ (3)

where E_di_ is direct energy input (MJ·ha^−1^), and E_indi_ is indirect energy input (MJ·ha^−1^).

**Figure 6:**
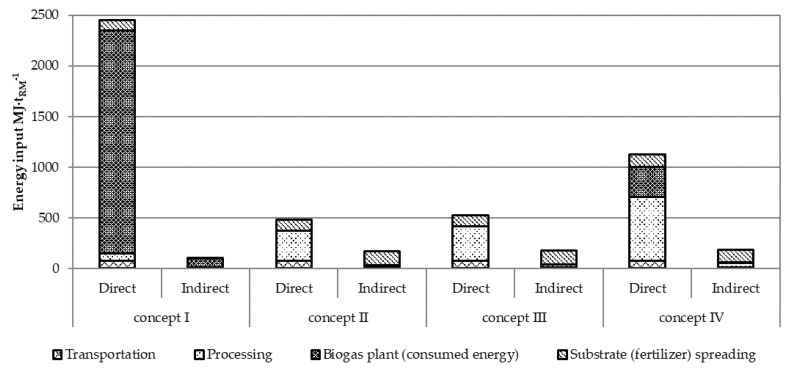
The direct and indirect energy input of zero-waste bio-refining of the fish co-stream.

The results obtained after recalculation for 1 t of fish co-stream shows that energy input at biogas plant was higher in concept I than in concept IV, see Figure 6.

In concept I, the mass of the fish co-stream was estimated to be 2400 t per year and 12,000 t per year of cattle manure. The feedstock conversion to biogas requires a total energy input of 2548 MJ·t_RM_^−1^ (of that 160 kWh·t_RM_^−1^ of electricity). After anaerobic treatment, the produced biogas was used for energy production. Additional processing of the fish co-stream (grinding, thermal treatment, and phase separation) is energy-intensive, and it required 82 MJ·t_RM_^−1^ in concept I, 326 MJ·t_RM_^−1^ in concept II, 377 MJ·t_RM_^−1^ in concept III, and 678 MJ·t_RM_^−1^ in concept IV. Energy generated in concept I was the highest among the analysed cases and reached 9992 MJ·t_RM_^−1^, but at the same time the energy input for the operation of the biogas plant, i.e., self-consuming, was 2196 MJ·t_RM_^−1^ and lowered the total output of useful energy. Concept IV had useful energy generated by the biogas plant of 1299 MJ·t_RM_^−1^, while self-consumption was 293 MJ·t_RM_^−1^.

In concept II, the mass of the fish co-stream (meal) was 379 t per year and was used as animal feed; therefore, no energy was produced in this case. Processing of the fish co-stream to animal feed required 326 MJ·t_RM_^−1^.

In concept III, the mass of the fish co-stream (meal) was 312 t per year and was processed to fish meal, oil, and proteins. Processing of the fish co-stream required 377 MJ·t_RM_^−1^.

**Figure 7:**
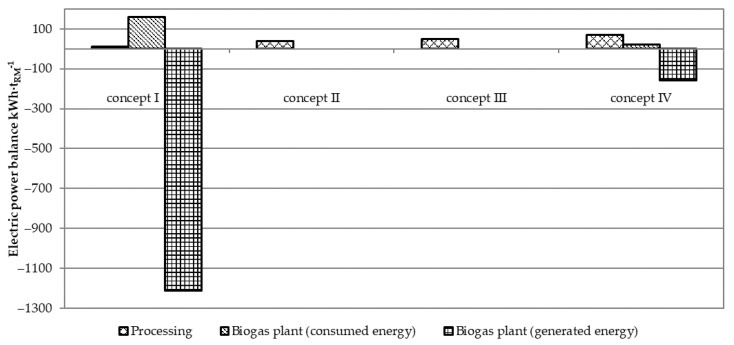
Electric power balance. Note: positive numbers indicate energy consumption while negative indicate energy generation.

After anaerobic treatment, the produced biogas was used for electricity production with a total generation of 1213 kWh·t_RM_^−1^ in concept I and 158 kWh·t_RM_^−1^ in concept IV (Figure 7). Internal usage in processing the fish co-stream and biogas plant operation reduced useful (ready to sell to the network) power to 1042 kWh·t_RM_^−1^ in concept I and 66 kWh·t_RM_^−1^ in concept IV. Concepts II and III consumed electric power for processing the fish co-stream, sequentially 41 kWh·t_RM_^−1^ and 51 kWh·t_RM_^−1^.

Transportation required the same amount of energy in all concepts due to the same mass to be transported and needed 82.1 MJ·t_RM_^−1^ in total.

**Figure 8:**
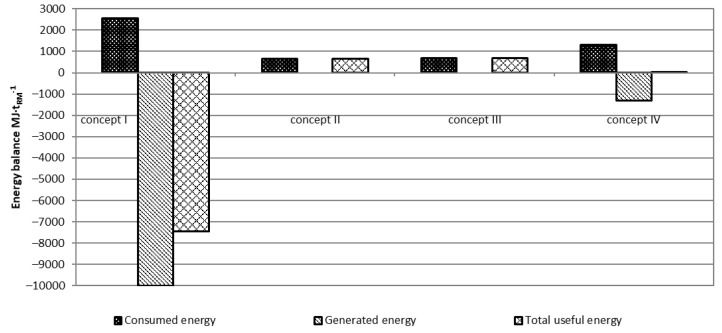
Total energy balance. Note: positive numbers indicate energy consumption while negative indicate energy generation.

By using a higher mass of fish co-stream for biogas generation despite higher usage of energy, the total useful energy increased as well (Figure 8). Only concept I and concept IV generated additional energy due to the biogas plant. The ratio of energy input/output shows that the higher and most efficient treatment of the fish co-stream was concept I when a high amount of fish co-streams was delivered directly to the biogas plant. In such a way the ratio was 3.9, while in concept IV it was 1.0 only.

### 3.3. Economic Feasibility

The variable costs (raw materials, utilities, logistics, labour, and maintenance) and fixed costs (depreciation and other fixed costs) were calculated according to [61]. The economic feasibility of four technological concepts for zero-waste processing of fish co-streams is evaluated in terms of return on investment (ROI), expressed as

(4) $$\mathrm{ROI}=\frac{\mathrm{Annual}\;\mathrm{net}\;\mathrm{profit}}{\mathrm{Investment}\;\mathrm{costs}}$$

Certain uncertainties are involved in the early stages of concept design. Consequently, stochastic simulation, here Monte Carlo simulation, was applied to assess these uncertainties. Applied prices are presented in Table 4 and obtained from [49]. The conversion used in this study was €1.0 EUR is $1.1 USD.

The estimates of capital costs for the four presented technological concepts are given in Table 5. The estimates of capital costs were based on the literature values [49,62] and step-counting method [61].

Other relevant cost factors are listed in Table 6. The average salary of 65,000 USD was assumed with additional 40% indirect costs on top. Maintenance and repairs were assumed as 2.5% of the original investment. Other indirect costs were 1% of the investment. For logistics, a cost of 0.1 USD·t^−1^·km^−1^ was assumed.

Total annual production costs are listed in Table 7 and total revenues in Table 8. It is seen that raw materials and capital costs were the main cost factors here. Largest revenues were gained for premium quality oil and fish protein hydrolysate (concept IV). Revenues from the energy were at the same level or higher than used for energy utilities in the fish co-stream processing plant.

The profitability of four different concepts is given in Table 9. If the fish co-stream is used for plain biogas production (I), it is seen that the production was not profitable. All other three concepts generated positive cash flow, and the two-stage process gained almost $6·10^6^ USD annually. Return on investment was also positive for concepts II, III, and IV, see Table 9.

**Figure 9:**
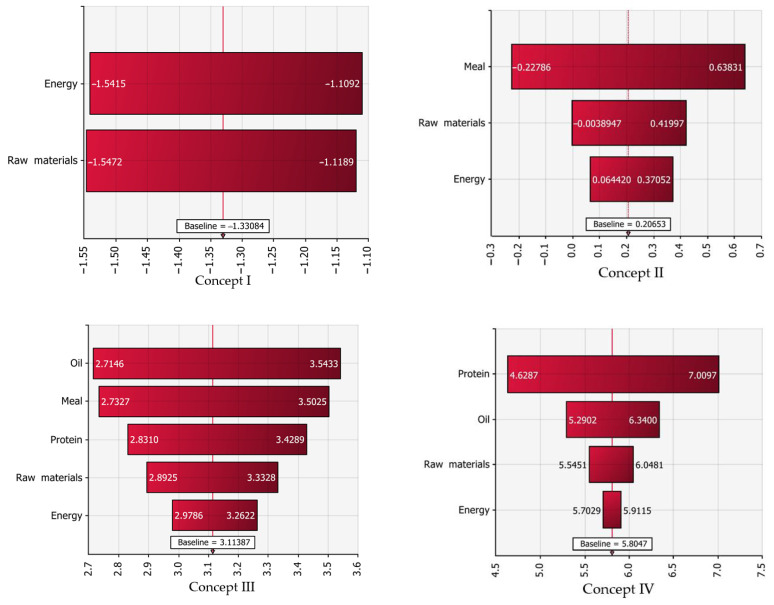
Sensitivity analysis of profitability. Four different fish co-stream processing technologies presented here. Baseline refers to annual profit.

A sensitivity analysis was conducted to illustrate the most critical factor of economic evaluation. The prices of raw materials, energy, fish oil, fish protein, and fish meal were varied ±20%. (For example, the prices of mineral fertilisers and formic acid were considered to change according to variations in energy price.) Sensitivity analysis was based on the Monte Carlo simulations, with 10,000 iterations. Results of four different concepts are shown in Figure 9.

Biogas production (I) was equally sensitive to the revenues from the produced energy and the fish residue price. Fish silage process (II) was critical for the revenues of liquid silage. Thermal processing of fish-side stream (III) indicated that the prices of fish oil and dried fish meal were the most critical aspects. On the other hand, the two-stage thermal-enzymatic processing of fish co-stream (IV) generated significant revenues from the high-quality fish protein hydrolysate. This factor is the most critical when profitability is considered.

## 4. Discussion

An energetic and economic evaluation of four different fish co-stream processing technologies showed a clear contradiction between energy production and economic feasibility. The largest amount of energy is produced when the entire fish co-stream is considered as residue and transported to farms for biogas production (I). This concept also allowed omitting the mineral fertilisers entirely, as combined fish co-stream and manure provided enough K, N, and P for winter barley cultivation.

On the other hand, the two-stage processing of the fish-side stream (IV) utilising valuable marine biomass by producing premium fish oil and high-quality fish protein hydrolysate was a most economically feasible concept. Here, the solid residue was transported to farms and utilised in smaller-scale biogas production. The annual revenues of this new process were significantly higher than the thermal processing of fish co-stream (III) or traditional fish silage process (II). Naturally, this new concept benefits the assumed high price of the better-quality oil and fish protein hydrolysate. If food-grade quality is not achieved, the profitability is lower, as the sensitivity analysis showed.

Biogas production (I) required a large amounts of the fish co-stream assumed in this study. If very low-quality fish co-streams would be considered, the production of feed and food production would not be an option. A biogas plant could be considered an elegant way to handle this solid waste residue and to utilise the organic matter for biogas and inorganic components for fertilisers.

When the two-stage concept is considered, the solid fraction could be utilised, at least partly for meal production, and an even higher profitability for concept (IV) would be achieved. However, the solid fraction is a possible source of marine phospholipids, and consequently, additional processing might be applied. After the extraction of these valuable components, the biogas production of remaining residue might be the best option.

Battery limits of this study are defined in such a way that fish co-stream processing plant, biogas production, and farms are considered as a single unity. In reality, these plants would form a value chain or network where intermediates (such as fish residue) are purchased at a certain price. This analysis is outside the scope of this study, but as positive cash flow is generated across the studied battery limit, it might be possible to define prices for intermediates where both fish co-stream operator and farms are gaining profits.

Certain uncertainties are involved in the early stages of concept design, the availability of data is limited, and simplified models may be used. The performed economic analysis evaluates the key cost items to focus on future development stages and gives an economic ranking of the proposed fish co-stream process options. The obtained information can be used to compare process options, select concepts for further optimisation and more detailed design, and support the decision-making process.

## 5. Conclusions

This study reviewed four different technological concepts for zero-waste processing of fish (salmon) co-streams. Fish co-stream processing plants, production of biogas from fish residues, and utilising digestate as fertilisers were considered in this study. Energetic and economic analyses were conducted.

From an energetic point of view, the processing of fish residue to biogas and fertilisers is the most tempting option. However, valuable marine components will not be recovered in this case. Technologies of biogas and fertiliser production should be used when obtained co-streams are low quality, and the extraction of other valuable components is not possible. If such a stream is available, this concept utilises the organic fraction of residue for energy production, recycles the inorganic components as fertilisers, and reduces the energy need and amount of purchased mineral fertiliser in farms.

Two-stage processing of fish co-streams leads to the recovery of both high-quality marine oil and proteins and shows the largest profitability and return on investment during the economic analysis. It is a more tempting option than the currently used thermal treatment or traditional silage processes. The possibility of producing high-quality and food-grade fish protein hydrolysate is the biggest benefit here. Conclusions indicate contradiction between energy production and economic feasibility of the evaluated processes. Quality of co-streams should be defined as the main indicator for making decisions in industries utilising fish co-streams. Chemical composition of by-products would slightly change the outcomes and yields of the presented technologies, and this would not have a significant influence on the final outcome.

Concepts studied herein are examples of zero-waste processing of bio-products and illustrate the possibilities and benefits of fully utilising whole fish, in form of various products, such as fillets, oil, and protein, for fertilisers and energy production.

## Figures and Tables

**Table 1:** Material and energy balance of the fish processing plant.

Raw-Material/Utility	Unit ^a^	I	II	III	IV
Input					
Fish co-stream	(kg·t_RM_^−1^)	1000	1000	1000	1000
Formic acid	(kg·t_RM_^−1^)		6.7		
Enzyme	(kg·t_RM_^−1^)				1
Steam	(kg·t_RM_^−1^)		0.5	0.7	1.3
Electricity	(kWh·t_RM_^−1^)		30	40	60
Water	(m^3^·t_RM_^−1^)				0.4
Output					
Premium oil	(kg·t_RM_^−1^)				175
Oil	(kg·t_RM_^−1^)			187	46
Fish protein concentrate	(kg·t_RM_^−1^)			189	
Fish protein hydrolysate	(kg·t_RM_^−1^)				171
Meal	(kg·t_RM_^−1^)			158	
Silage	(kg·t_RM_^−1^)		1000		
Residue	(kg·t_RM_^−1^)	1000			130

^a^ RM refers to fish co-stream as raw material.

**Table 2:** Material and energy balance of the biogas plant.

Raw-Material/Utility	Unit ^a^	I	II	III	IV
Input					
Fish residue	(kg·t_RM_^−1^)	1000			130
Manure	(kg·t_RM_^−1^)	5000			650
Output					
Heat	(kWh·t_RM_^−1^)	1562			203
Electricity	(kWh·t_RM_^−1^)	1213			158
Fertilisers	(kg·t_RM_^−1^)	5168			662

^a^ RM refers to fish co-stream as raw material.

**Table 3:** Chemical composition of salmon backbones and cattle manure.

Parameter	Salmon Backbones after Filleting	Liquid Cattle Manure
Total solids (TS) wt.%	41.6	6.3
In total solids:		
Volatile solids (VS) wt.%	92.5	72.0
Organic carbon (C) wt.%	69.8	39.4
Total nitrogen (N) mg·kg^−1^	62,357	59,344
Total phosphorus (P) mg·kg^−1^	12,912	10,376
Total potassium (K) mg·kg^−1^	5583	60,414
Calcium (Ca) wt.%	2.3	3.2
Magnesium (Mg) wt.%	0.06	1.01
Copper (Cu) mg·kg^−1^	8	93
Zink (Zn) mg·kg^−1^	32	267
Manganese (Mn) mg·kg^−1^	6	235
Iron (Fe) mg·kg^−1^	20	1178
Boron (B) mg·kg^−1^	18	43
Sulphur (S) mg·kg^−1^	294	4256
Fat wt.%	54.2	4.1

**Table 4:** Applied prices.

Product	Unit	Value
Fish co-stream	(USD·t_RM_^−1^)	120
Formic acid	(USD·t_RM_^−1^, 80 v.%)	1000
Enzyme	(USD·kg^−1^)	20
Steam	(USD·t^−1^)	20
Electricity	(USD·MWh^−1^)	100
Water	(USD m^−3^)	0.1
Premium fish oil	(USD·t^−1^)	1350
Fish oil	(USD·t^−1^)	1200
Fish protein hydrolysate	(USD·t^−1^)	3900
Fish protein concentrate	(USD·t^−1^)	900
Meal	(USD·t^−1^)	1350
Silage	(USD·t^−1^)	240
Mineral fertilisers	(USD·t^−1^)	500

**Table 5:** Investment costs.

Fish Co-Stream Processing	Unit	Concept I	Concept II	Concept III	Concept IV
Capacity	(t_RM_·a^−1^)	10,000	10,000	10,000	10,000
Investment	(USD)		1.0 × 10^6^	7.4 × 10^6^	9.5 × 10^6^
Biogas plant					
Fish residue	(t_RM_·a^−1^)	10,000			1300
Manure	(t_RM_·a^−1^)	50,000	50,000	50,000	50,000
Biogas capacity	(t_RM_·a^−1^)	60,000			7800
Biogas capacity/farm	(t_RM_·a^−1^)	15,000			7800
Number of biogas plants		4			1.0
Investment	(USD per farm)	2.6 × 10^6^			1.8 × 10^6^
Total investments	(USD)	10.4 × 10^6^	1.0 × 10^6^	7.4 × 10^6^	11.3 × 10^6^

**Table 6:** Other costs.

Other Costs	Unit	Concept I	Concept II	Concept III	Concept IV
Operators		2	1	3	5
Salary	(USD·a^−1^)	65,000	65,000	65,000	65,000
Indirect costs ^a^	(%)	40%	40%	40%	40%
Maintenance ^b^	(%)	2.5%	2.5%	2.5%	2.5%
Depreciation time	(a)	15	15	15	15
Other indirect ^b^	(%)	1.0%	1.0%	1.0%	1.0%
Logistics	(USD·t^−1^·km^−1^)	0.1	0.1	0.1	0.1
Distance	(km)	100	100	100	100

^a^ % of salary; ^b^ % of the original investment.

**Table 7:** Costs.

Costs	Unit	Concept I	Concept II	Concept III	Concept IV
Raw materials	(USD·a^−1^)	1.2 × 10^6^	1.3 × 10^6^	1.2 × 10^6^	1.4 × 10^6^
Utilities	(USD·a^−1^)		0.1 × 10^6^	0.2 × 10^6^	0.3 × 10^6^
Labour	(USD·a^−1^)	0.2 × 10^6^	0.1 × 10^6^	0.3 × 10^6^	0.5 × 10^6^
Logistics	(USD·a^−1^)	0.1 × 10^6^			0.01 × 10^6^
Maintenance	(USD·a^−1^)	0.3 × 10^6^	0.03 × 10^6^	0.2 × 10^6^	0.3 × 10^6^
Depreciation	(USD·a^−1^)	0.7 × 10^6^	0.1 × 10^6^	0.5·× 10^6^	0.8 × 10^6^
Other indirect	(USD·a^−1^)	0.1 × 10^6^	0.01 × 10^6^	0.1 × 10^6^	0.1 × 10^6^
Mineral fertilisers	(USD·a^−1^)	0.0 × 10^6^	0.6 × 10^6^	0.6 × 10^6^	0.5 × 10^6^
Total	(USD·a^−1^)	2.5 × 10^6^	2.2 × 10^6^	3.0·× 10^6^	3.9 × 10^6^

**Table 8:** Revenues.

Revenues	Unit	Concept I	Concept II	Concept III	Concept IV
Oil	(USD·a^−1^)			2.3 × 10^6^	2.9 × 10^6^
Protein	(USD·a^−1^)			1.7 × 10^6^	6.6 × 10^6^
Meal	(USD·a^−1^)		2.4 × 10^6^	2.2 × 10^6^	
Energy	(USD·a^−1^)	1.2 × 10^6^			0.2 × 10^6^
Total	(USD·a^−1^)	1.2 × 10^6^	2.4 × 10^6^	6.2 × 10^6^	9.7 × 10^6^

**Table 9:** Profit.

Indicator	Unit	Concept I	Concept II	Concept III	Concept IV
Profit	(USD·a^−1^)	−1.3 × 10^6^	0.2 × 10^6^	3.1 × 10^6^	5.8 × 10^6^
ROI	(%)	−13%	21%	42%	51%

## Data Availability

All data generated or analysed during the study are included in this published article. The datasets used and/or analysed during the present study are available from the corresponding author upon reasonable request.
